# Risk factors related to perioperative systemic complications and mortality in elderly patients with osteoporotic vertebral fractures—analysis of a large national inpatient database

**DOI:** 10.1186/s13018-020-02050-5

**Published:** 2020-11-10

**Authors:** Shingo Morishita, Toshitaka Yoshii, Atsushi Okawa, Hiroyuki Inose, Takashi Hirai, Masato Yuasa, Kiyohide Fushimi, Takeo Fujiwara

**Affiliations:** 1 Department of Orthopedic Surgery, Tokyo Medical and Dental University Graduate School of Medicine, 1-5-45 Yushima, Bunkyo-ku, Tokyo, Japan; 2 Department of Health Policy and Informatics, Tokyo Medical and Dental University Graduate School of Medicine, Tokyo, Japan; 3 Department of Global Health Promotion, Tokyo Medical and Dental University Graduate School of Medicine, Tokyo, Japan

**Keywords:** Perioperative complications, Mortality, Fusion surgery, Osteoporotic vertebral fractures, Nationwide Inpatient Database

## Abstract

**Background:**

The surgical treatment of osteoporotic vertebral fractures (OVF) is generally associated with a high risk of complications due to an aging population with osteoporosis; however, the detailed risk factors for systemic complications and mortality have not been clarified. We evaluated the risk factors for systemic complications and mortality in surgically treated OVF patients using a large national inpatient database.

**Methods:**

Patients over 65 years old who were diagnosed with OVF and received either anterior fusion (AF) or posterior fusion (PF), from 2012 to 2016, were extracted from the diagnosis procedure combination (DPC) database. In each of the perioperative systemic complications (+) or (−) group, and the in-hospital death (+) or (−) group, we surveyed the various risk factors related to perioperative systemic complications and in-hospital death.

**Results:**

The significant factors associated with systemic complications were older age (OR 1.38, 95% CI 1.09–1.74), a lower activity of daily living score upon admission (OR 1.52, 95%CI 1.19–1.94), atrial fibrillation (OR 2.14, 95%CI 1.25–3.65), renal failure (OR 2.29, 95%CI 1.25–4.20), and surgical procedure (AF, OR 1.73, 95%CI 1.35–2.22). The significant explanatory variables for in-hospital death were revealed to be male sex (OR 3.26, 95%CI 1.20–8.87), a lower body mass index (OR 3.97, 95%CI 1.23–12.86), unscheduled admission (OR 3.52, 95%CI 1.17–10.63), atrial fibrillation (OR 8.31, 95%CI 2.25–30.70), renal failure (OR 7.15, 95%CI 1.32-38.77), and schizophrenia (OR 8.23, 95%CI 1.66–42.02).

**Conclusions:**

Atrial fibrillation and renal failure as preoperative comorbidities were common factors between perioperative systemic complications and mortality in elderly patients for OVF.

**Supplementary Information:**

The online version contains supplementary material available at 10.1186/s13018-020-02050-5.

## Introduction

Thoracolumbar osteoporotic vertebral fractures (OVF) generally develop due to bone fragility, especially in elderly patients [1], and OVF are the most common type of fragile fractures [2]. The inducing mechanism of this type of fracture in elderly people is slight injury, such as a fall from a standing height [3]. OVF are deeply associated with difficulties in terms of activities of daily living (ADL) and poor quality of life (QOL) and high risks of hospitalization and mortality [4]. Due to the aging of the population, the prevalence of osteoporosis has increased markedly, resulting in increases in the number of patients with OVF [5, 6]. The increase in OVF and the subsequent high mortality will become serious socioeconomic problems [7].

OVF patients are usually prescribed conservative and nonsurgical treatments consisting of pain medication, bed rest, physiotherapy, and bracing, and most patients with OVF respond well to conservative management [8, 9]. This type of fracture occasionally requires operative treatment because of neurological dysfunction or kyphotic malalignment after severe vertebral collapse and/or instability [10, 11]. Several studies have investigated surgical methods for the treatment of OVF and the functional outcomes of each method [12, 13]. However, few studies have focused on perioperative complications with large sample sizes. As the surgical treatment of OVF generally has a high risk of complications due to the increase in age in the population with osteoporosis, it is quite important to clarify the detailed risk factors for systemic complications and mortality in patients undergoing surgery. Thus, in this study, we investigated a large national inpatient database that included numerous elderly patients undergoing surgical treatment for OVF and evaluated the perioperative complications, mortality, and risk factors.

## Materials and methods

### Data sources

The retrospective study was conducted using the diagnosis procedure combination (DPC), which was developed by the Ministry of Health, Labor and Welfare in Japan. All 82 academic and voluntary general hospitals in Japan participate in the database [14]. All patient data in this study were obtained from the multi-institutional DPC database as stated in previous literature [15–18]. This database contains the following items: age, sex, body mass index (BMI), smoking index, admission type, emergency transport, hospital type, ADL scores for admission and discharge by the Ministry of Health, surgical procedure, diagnosis with International Classification of Diseases, Tenth Revision (ICD-10) codes, comorbidities at admission and complications after admission, and in-hospital deaths. The database contains a separate registry of comorbidities at admission and postoperative complications. The institutional ethical committee at Tokyo Medical and Dental University permitted us to use all data in the DPC database.

### Patient selection and data extraction

Patients over 65 years old and who were diagnosed with thoracic (ICD-10 code, S2200) or lumbar vertebral fracture (S3200) and osteoporosis (M800-805, 808-816, 818, 819) were eligible for inclusion in the DPC database. We selected patients who received operative treatment, either anterior fusion (AF, K142-1 according to the Japanese original surgical code; K-code) or posterior fusion (PF, K142-2), from April 1, 2012, to March 31, 2016. The use of anterior and posterior surgery upon the first admission was considered AF. Patients excluded from the current study were as follows: (1) patients less than 65 years of age, (2) patients without osteoporosis, (3) patients who received only vertebroplasty without spinal fusion (e.g., balloon kyphoplasty), (4) infectious spondylitis including pyogenic and tuberculosis, (5) metastatic or primary spinal tumors. Patient comorbidities at admission extracted in the current study were the following: diabetes mellitus (ICD-10 codes: E10-14), cardiovascular disease (I200, 201, 208-214, 219-221, 228, 229, 238), cardiac failure (I110, I500, 501, 509), atrial fibrillation (I48), hypertension (I10, 15), cerebrovascular disease (I614, 619, I630-639), chronic obstructive pulmonary disease (J441, 448, 449), pneumonia (J13, 14, 150-159, J180-182, 188, 189, J690, J958), renal failure (N17-19, N289, I120), hepatic failure (K704, 711, 719, 720, 729, 769), gastric ulcer and hemorrhage (K250-270, K279, K922), malignancy (C00-97), rheumatoid arthritis (M069), dementia (F000-002, 009, 010-012, 019, 03, G300-301, 308-309), depression (F313-315, 318-323, 328-334, 339), and schizophrenia (F200-209). Included perioperative systemic complications after admission were the following: cardiovascular disease, cardiac failure, atrial fibrillation, cerebrovascular disease, respiratory failure (J959-961, 969), pneumonia, dysphagia (K918, R13), renal failure, hepatic failure, gastric ulcer and hemorrhage, deep venous thrombosis (I801, 802, 828), pulmonary embolism (I269), sepsis (A394, 400-403, 409-415, 418, 419), delirium (F050, 051, 059), urinary tract infection (N390, T835), chylothorax (I898, S278, T812), hemothorax (J942, S271-272), pneumothorax (J930-931, 938-939, S270, T812), pleurisy (J90, R091, T812), and pyothorax (J869). We listed reoperations for systemic complications. The listed reoperations were as follows: cardiac (K codes; K538-605), vascular (K606-619, 621-623), pulmonary (K507-519), thoracic (K477-504), cerebral (K145-181), gastric (K520-533, 630-668), liver, gallbladder, pancreas (K669-709), and vena cava filter (K620), tracheoplasty (K403), or tracheostomy (K386).

### Statistical analysis

First, all patients in this study were divided into either the perioperative systemic complications (+) or (−) group during hospitalization. In the two groups, we compared the following parameters: age, sex, BMI, smoking index, emergency transport, hospital type, ADL score upon admission, surgical procedure, and preoperative comorbidities between the complication (+) group and the complication (−) group. Fisher’s exact test and the chi-square test were used to compare proportions of categorical variables. All continuous variables were converted into categorical variables. We further performed multivariate logistic regression analysis to investigate the risk factors related to perioperative systemic complications using the explanatory variables that were significant in the univariate analysis as *P* < 0.05. Second, all patients in this study were also divided into either the in-hospital death (+) or (−) group. We similarly conducted univariate analysis to reveal the risk factors for hospital mortality in this study between the in-hospital death (+) group and the in-hospital death (−) group. Multivariate logistic regression analysis was also performed using the variables with *P* < 0.05 in the univariate analysis. All statistical analyses were performed with Stata/MP version 14 (StataCorp, College Station, TX, USA), and *P* < 0.05 was considered statistically significant. We also calculated variance inflation factor (VIF) for variables that seemed to influence each other (e.g., cardiac failure and atrial fibrillation), taking multicollinearity into account. As the VIF was below 10, we considered the effect of multicollinearity of the explanatory variables to be small.

## Results

First, Table 1 describes whole demographics of total 2446 patients in this study. In total, the average age was 77.4 years old, BMI was 22.2 kg/m^2^, scheduled admission was 56.4%, emergency transport was 16.3%, and academic hospital was 5.4%. The common preoperative comorbidities were, in order of descending prevalence, hypertension (28.3%), diabetes mellitus (14.5%), and cardiovascular disease (5.6%).

**Table 1:** Characteristics of total patients

	Total patient (*N* = 2446)
**Age (years)**	77.4 (5.9)
**BMI (kg/m**^**2**^**)**	22.2 (3.7)
**Smoking index**	1011 (2889)
**Admission type**	
Scheduled	1379 (56.4%)
Unscheduled	1067 (43.6%)
**Emergency transport**	
Yes	400 (16.3%)
No	2046 (83.7%)
**Hospital type**	
Academic	131 (5.4%)
Non-academic	2315 (94.6%)
**ADL score for admission (points)**	10.5 (7.6)
**Preoperative comorbidities**	
Diabetes mellitus	354 (14.5%)
Cardiovascular disease	136 (5.6%)
Cardiac failure	76 (3.1%)
Atrial fibrillation	63 (2.6%)
Hypertension	691 (28.3%)
Cerebrovascular disease	34 (1.4%)
Chronic obstructive pulmonary disease	11 (0.5%)
Pneumonia	7 (0.3%)
Renal failure	49 (2.0%)
Hepatic failure	61 (2.5%)
Gastric ulcer and hemorrhage	90 (3.7%)
Malignancy	90 (3.7%)
Rheumatoid arthritis	102 (4.2%)
Dementia	49 (2.0%)
Depression	82 (3.4%)
Schizophrenia	46 (1.9%)

Continuous data are presented as the mean (standard division). Categorical data were presented as *n* (%)

*BMI* body mass index, *ADL* activity of daily living

Table 2 shows the demographics of all patients in terms of the perioperative systemic complications (+) or (−) group. Whole systemic complications were observed in 498 patients, which accounted for 20% of the total 2446 patients. The patients in the complication (+) group were characterized as follows: there were fewer elderly patients (over 85 years old, complication (+)/(−) group: 13.3%/11.6%, *P* < 0.001), more were unscheduled at admission (48.4%/42.4%, *P* = 0.016), they had a lower ADL score at admission (0 to 10 points: 47.8%/40.0%, *P* < 0.001), and more patients received AF (23.3%/16.4%, *P* < 0.001) (Table 2).

**Table 2:** Patient characteristics between systemic complication (+) and (−)

	Complication (−) (*N* = 1948)	Complication (+) (*N* = 498)	*P* value
**Age (years)**			0.012*
65 to 74	627 (32.2%)	126 (25.3%)	
75 to 84	1094 (56.2%)	306 (61.4%)	
Over 85	227 (11.6%)	66 (13.3%)	
**Sex**			0.06
Male	469 (24.1%)	140 (28.1%)	
Female	1479 (75.9%)	358 (71.9%)	
**BMI (kg/m**^**2**^**)**			0.16
Less than 18.5	272 (14.0%)	76 (15.2%)	
18.5 to 22.9	871 (44.7%)	207 (41.6%)	
23.0 to 24.9	349 (17.9%)	78 (15.7%)	
25.0 to 29.9	322 (16.5%)	88 (17.7%)	
Over 30.0	51 (2.6%)	16 (3.2%)	
Unknown	83 (4.3%)	33 (6.6%)	
**Smoking index**			0.82
0	1899 (78.2%)	13 (72.2%)	
1 to 999	227 (9.4%)	2 (11.1%)	
Over 1000	302 (12.4%)	3 (16.7%)	
**Admission type**			0.016*
Scheduled	1122 (57.6%)	257 (51.6%)	
Unscheduled	826 (42.4%)	241 (48.4%)	
**Emergency transport**			0.31
Yes	311 (16.0%)	89 (17.9%)	
No	1637 (84.0%)	409 (82.1%)	
**Hospital type**			0.46
Academic	101 (5.2%)	30 (6.0%)	
Non-academic	1847 (94.8%)	468 (94.0%)	
**ADL score for admission (points)**			< 0.001***
0 to 10	780 (40.0%)	238 (47.8%)	
11 to 20	841 (43.2%)	165 (33.1%)	
Unknown	327 (16.8%)	95 (19.1%)	
**Surgical procedure**			< 0.001***
Anterior fusion	319 (16.4%)	116 (23.3%)	
Posterior fusion	1629 (83.6%)	382 (76.7%)	
**Preoperative comorbidities**			
Diabetes mellitus	270 (13.9%)	84 (16.9%)	0.09
Cardiovascular disease	100 (5.1%)	36 (7.2%)	0.07
Cardiac failure	60 (3.1%)	16 (3.2%)	0.88
Atrial fibrillation	41 (2.1%)	22 (4.4%)	0.004**
Hypertension	541 (27.8%)	150 (30.1%)	0.30
Cerebrovascular disease	27 (1.4%)	7 (1.4%)	0.97
Chronic obstructive pulmonary disease	8 (0.4%)	3 (0.6%)	0.57
Pneumonia	6 (0.3%)	1 (0.2%)	0.69
Renal failure	32 (1.6%)	17 (3.4%)	0.012*
Hepatic failure	44 (2.3%)	17 (3.4%)	0.14
Gastric ulcer and hemorrhage	75 (3.9%)	15 (3.0%)	0.38
Malignancy	72 (3.7%)	18 (3.6%)	0.93
Rheumatoid arthritis	74 (3.8%)	28 (5.6%)	0.07
Dementia	39 (2.0%)	10 (2.0%)	0.99
Depression	66 (3.4%)	16 (3.2%)	0.85
Schizophrenia	33 (1.7%)	13 (2.6%)	0.18

Significant values are given as follows: *P < 0.05, **P < 0.01, ***P < 0.001

*BMI* body mass index, *ADL* activity of daily living

We also show the risk factors for perioperative systemic complications according to the multivariable analysis in Table 3. The significant factors proven to be associated with systemic complications were older age (75 to 84 years old, odds ratio [OR] 1.38, 95% confidence interval [CI] 1.09–1.74, *P* = 0.007), a lower ADL score upon admission (0 to 10 points, OR 1.52, 95%CI 1.19–1.94, *P* = 0.001), atrial fibrillation (OR 2.14, 95%CI 1.25–3.65, *P* = 0.006), renal failure (OR 2.29, 95%CI 1.25–4.20, *P* = 0.007), and surgical procedure (AF, OR 1.73, 95%CI 1.35–2.22, *P* < 0.001) (Table 3).

**Table 3:** Risk factor for perioperative systemic complications with multivariable logistic regression analysis

	OR	*P* value	95%CI
**Age (years)**			
65 to 74	Reference		
75 to 84	1.38	0.007**	1.09–1.74
Over 85	1.40	0.054	0.99-1.97
**Admission type**			
Scheduled	Reference		
Unscheduled	1.16	0.18	0.93–1.45
**ADL score for admission (points)**			
0 to 10	1.52	0.001**	1.19–1.94
11 to 20	Reference		
Unknown	1.49	0.008**	1.11–1.99
**Preoperative comorbidities**			
Atrial fibrillation	2.14	0.006**	1.25–3.65
Renal failure	2.29	0.007**	1.25–4.20
**Surgical procedure**			
Posterior fusion	Reference		
Anterior fusion	1.73	< 0.001***	1.35–2.22

Significant values are given as follows: **P < 0.01, ***P < 0.001

*ADL* activity of daily living, *OR* odds ratio, *CI* confidence interval

**Fig. 1:**
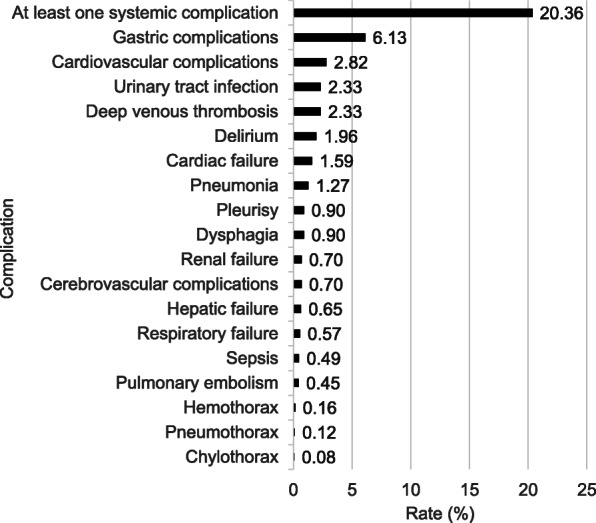
List of systemic complication and complication rate

Figure 1 shows a list of systemic complications in this study. The incidence of systemic complications was approximately 20%, including gastrointestinal complications (6.1%), cardiovascular complications (2.8%), urinary tract infections (2.3%), and deep vein thrombosis (2.3%) (Fig. 1). We further listed reoperations for systemic complications in each organ (Additional Table 1).

Table 4 shows the demographic data for a total of 2446 patients, including 18 deceased patients. The patients in the in-hospital death (+) group had the following characteristics: there were more males (in-hospital death (+)/(−): 61.1%/24.6%, *P* < 0.001), they had a lower BMI (less than 18.5 kg/m^2^: 44.4%/14.0%, *P* = 0.001), more were unscheduled at admission (72.2%/43.4%, *P* = 0.014), more had cardiac failure (11.1%/3.1%, *P* = 0.009), more had atrial fibrillation (22.2%/2.4%, *P* < 0.001), more had renal failure (11.1%/1.9%, *P* = 0.006), and more had schizophrenia (11.1%/1.8%, *P* = 0.004) compared with patients in the in-hospital death (−) group (Table 4).

**Table 4:** Patient characteristics between in-hospital death (+) and (−)

	Death (−) (*N* = 2428)	Death (+) (*N* = 18)	*P* value
**Age (years)**			0.25
65 to 74	750 (30.9%)	3 (0.4%)	
75 to 84	1389 (57.2%)	11 (0.8%)	
Over 85	289 (11.9%)	4 (1.4%)	
**Sex**			< 0.001***
Male	598 (24.6%)	11 (61.1%)	
Female	1830 (75.4%)	7 (38.9%)	
**BMI (kg/m**^**2**^**)**			0.001**
Less than 18.5	340 (14.0%)	8 (44.4%)	
18.5 to 22.9	1073 (44.2%)	5 (27.8%)	
23.0 to 24.9	426 (17.5%)	1 (5.6%)	
25.0 to 29.9	409 (16.9%)	1 (5.6%)	
Over 30.0	67 (2.8%)	0 (0%)	
Unknown	113 (4.6%)	3 (16.6%)	
**Smoking index**			0.82
0	1899 (78.2%)	13 (72.2%)	
1 to 999	227 (9.4%)	2 (11.1%)	
Over 1000	302 (12.4%)	3 (16.7%)	
**Admission type**			0.014*
Scheduled	1374 (56.6%)	5 (27.8%)	
Unscheduled	1054 (43.4%)	13 (72.2%)	
**Emergency transport**			0.19
Yes	395 (16.3%)	5 (27.8%)	
No	2033 (83.7%)	13 (72.2%)	
**Hospital type**			0.31
Academic	131 (5.4%)	0 (0%)	
Non-academic	2297 (94.6%)	18 (100%)	
**ADL score for admission (points)**			0.26
0 to 10	1008 (41.5%)	10 (55.6%)	
11 to 20	1002 (41.3%)	4 (22.2%)	
Unknown	418 (17.2%)	4 (22.2%)	
**Surgical procedure**			0.62
Anterior fusion	431 (17.8%)	4 (22.2%)	
Posterior fusion	1997 (82.2%)	14 (77.8%)	
**Preoperative comorbidities**			
Diabetes mellitus	350 (14.4%)	4 (22.2%)	0.35
Cardiovascular disease	134 (5.5%)	2 (11.1%)	0.30
Cardiac failure	74 (3.1%)	2 (11.1%)	0.049*
Atrial fibrillation	59 (2.4%)	4 (22.2%)	< 0.001***
Hypertension	691 (28.5%)	0 (0%)	0.008**
Cerebrovascular disease	33 (1.4%)	1 (5.6%)	0.13
Chronic obstructive pulmonary disease	11 (0.5%)	0 (0%)	0.78
Pneumonia	7 (0.3%)	0 (0%)	0.82
Renal failure	47 (1.9%)	2 (11.1%)	0.006**
Hepatic failure	60 (2.5%)	1 (5.6%)	0.40
Gastric ulcer and hemorrhage	90 (3.7%)	0 (0%)	0.41
Malignancy	89 (3.7%)	1 (5.6%)	0.67
Rheumatoid arthritis	101 (4.2%)	1 (5.6%)	0.77
Dementia	48 (2.0%)	1 (5.6%)	0.28
Depression	81 (3.3%)	1 (5.6%)	0.60
Schizophrenia	44 (1.8%)	2 (11.1%)	0.004**

Significant values are given as follows: *P < 0.05, **P < 0.01, ***P < 0.001

*BMI* body mass index, *ADL* activity of daily living

Finally, we demonstrate the risk factors for in-hospital death according to multivariable logistic regression analysis during hospitalization in Table 5. The significant explanatory variables revealed by this analysis were male sex (OR 3.26, 95%CI 1.20–8.87; *P* = 0.021), lower BMI (less than 18.5 kg/m^2^, OR 3.97, 95%CI 1.23–12.86, *P* = 0.021), unscheduled at admission (OR 3.52, 95%CI 1.17–10.63, *P* = 0.026), atrial fibrillation (OR 8.31, 95%CI 2.25–30.70, *P* = 0.001), renal failure (OR 7.15, 95%CI 1.32-38.77, *P* = 0.023), and schizophrenia (OR 8.23, 95%CI 1.66–42.02, *P* = 0.010) (Table 5).

**Table 5:** Risk factor for in-hospital death with multivariable logistic regression analysis

	OR	P value	95%CI
**Sex**			
Female	Reference		
Male	3.26	0.021*	1.20–8.87
**BMI (kg/m**^**2**^**)**			
Less than 18.5	3.97	0.021*	1.23–12.86
18.5 to 22.9	Reference		
23.0 to 24.9	0.55	0.59	0.06–4.87
25.0 to 29.9	0.59	0.64	0.07–5.29
**Admission type**			
Scheduled	Reference		
Unscheduled	3.52	0.026*	1.17–10.63
**Preoperative comorbidities**			
Cardiac failure	3.42	0.16	0.61–19.14
Atrial fibrillation	8.31	0.001**	2.25–30.70
Renal failure	7.15	0.023*	1.32–38.77
Schizophrenia	8.23	0.010*	1.66–42.02

Significant values are given as follows: *P < 0.05, **P < 0.01

*BMI* body mass index, *OR* odds ratio, *CI* confidence interval

In summary, the risk factors for perioperative systemic complications were high age, low ADL score, comorbidities of atrial fibrillation and renal failure, and anterior surgery. The risk factors for death during hospitalization were male, low BMI, unscheduled admission, comorbidities of atrial fibrillation, renal failure, and schizophrenia. Atrial fibrillation and renal failure were risk factors for both postoperative systemic complications and in-hospital death.

## Discussion

We showed the risk factors for perioperative systemic complications and mortality in patients who underwent surgery for OVF using a large national inpatient database in Japan. The results obtained for the risk factors associated with systemic complications were older age, lower ADL score upon admission, atrial fibrillation, renal failure, and surgical procedure, i.e., AF. Moreover, we also revealed the risk factors significantly associated with in-hospital death for OVF: male sex, a lower BMI, unscheduled at admission, atrial fibrillation, renal failure, and schizophrenia. Among them, atrial fibrillation and renal failure were common factors for both mortality and perioperative systemic complications as preoperative comorbidities.

**Fig. 2:**
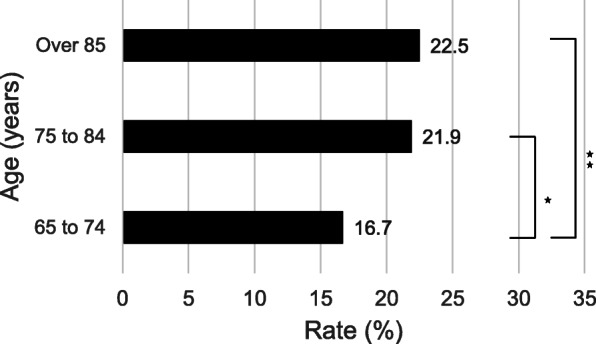
Systemic complication rate by age. Significant values are given as follows: *P < 0.05, **P < 0.01

As shown in Table 3 and Fig. 2, age was found to be one of the risk factors for perioperative systemic complications. The incidence of systemic complications increased with age and was particularly pronounced with age over 75 years (Fig. 2). Hamel et al. reported that 20% of older patients who underwent major noncardiac surgery experienced one or more postoperative complications, which supports our results (498 cases of total 2446 cases, with an incidence rate of 20.4%) [19]. Recent studies on perioperative complications after spine surgery have suggested an association with sarcopenia [20] and poor nutritional status [21] in aged patients. These factors may also be associated with postoperative complications and mortality in surgeries for OVF, although they were not evident in the current study because of the lack of detailed information in the database.

The results of this study showed that preoperative ADL was significantly associated with postoperative systemic complications with an OR of 1.52. Patients with lower ADL levels are often unable to walk independently and thus need to move with wheelchairs or are even bedridden due to their leg pain, numbness, and/or paralysis before the surgery. This condition can be associated with various systemic complications during the perioperative period, such as pneumonia [22], urinary tract infections due to catheter use [23], and pulmonary embolism [24, 25]. Therefore, it is important to consider surgical intervention before the patient’s functioning and ADL are seriously deteriorated due to OVF and vertebral collapse.

Anterior fusion is an effective and reasonable method that can reconstruct the anterior column and decompress neural tissue in the spinal canal. However, anterior fusion is associated with serious perioperative systemic complications, including pulmonary embolism [26], urinary tract infection [27], and pleurisy. Anterior surgery generally necessitates longer operative times with large quantities of blood loss, which results in the demand for transfusion more than posterior surgery [26]. The use of blood transfusion after spinal surgery is associated with high risks of urinary tract infection and other types of infection owing to some immunological problems [27]. Furthermore, the anterior surgical approach for accessing the thoracolumbar spine sometimes injures the diaphragm [28] and pleura [29], and this type of injury can be closely associated with respiratory complications. In the aging population, postoperative respiratory disturbance is sometimes serious and life-threatening. Thus, less-invasive procedures such as vertebroplasty and/or posterior percutaneous pedicle screw fixation [30, 31] should be alternatively considered if applicable to the patient’s condition.

A previous study revealed that impaired renal function, in particular, an estimated glomerular filtration rate (eGFR) of < 60 mL/min/1.73 m^2^, was a significant predictor for mortality after vertebral fracture [32]. Puvanesarajah et al. reported that elderly patients suffering from renal disease treated with lumbar fusion had 5.0 times increased mortality within 90 days of surgery compared to the control group [33]. They presumed that advanced renal disease was deeply associated with experiencing pulmonary embolism, myocardial infarction, pneumonia, respiratory failure, or cerebrovascular accident, which can lead to in-hospital death. Regarding cardiac problems, Fineberg et al. demonstrated the association between cardiac complications and lumbar spine surgeries and that cardiac events resulted in increased lengths of hospitalization and mortality [34]. Patients with atrial fibrillation have a significantly higher risk of perioperative mortality than patients with coronary artery disease undergoing noncardiac surgeries [35]. Atrial fibrillation is profoundly pertinent to thromboembolic events, which may lead to fatal hemodynamic decompensation due to the dysfunction of correct atrial contraction [36]. For elderly OVF patients, atrial fibrillation may be particularly relevant to mortality and complication factors according to our results.

Nakano et al. further indicated that male sex and low nutritional status may cause increased mortality in vertebral fracture patients, even in addition to low renal function [32]. Their logic supports our results, as male sex and low BMI were associated with in-hospital systemic complication events and mortality in patients with OVF who underwent surgical treatment. Another study confirmed that serum albumin concentration, which can reflect patients’ nutritional condition and BMI, was a significant predictor of mortality in elderly patients with fractures [37]. In particular, they established that the cut-off BMI value was 18.9 kg/m^2^.

Concerning the relationship between mortality and schizophrenia, the average life expectancy of patients with schizophrenia has been reported to be shorter than that of the general population, and they have a mortality risk that is two to three times that of the general population [38]. Another study reported that the higher in-hospital mortality in patients with schizophrenia may have resulted from impaired access to and low-quality care for primary disease due to psychiatric problems and unmeasured physical factors other than psychiatric factors [39]. Interestingly, in regard to unscheduled admission, recent studies discovered the possibility of an increased risk of death associated with hospital admission on the weekend [40, 41]. Thomas et al. found that fracture patients were more likely to die as an inpatient when admitted on the weekend [40]. In another paper, patients admitted on the weekend were significantly less likely to receive early spinal intervention [42]. These findings suggest that increased mortality with weekend admission may be explained by limited orthopedic resources, reductions in medical staff in hospital wards, and decreased access to diagnostic tools [40].

This study has several limitations. This national database cannot reflect clinically important findings since the database system was diagnosed with coding using ICD-10 codes without radiological findings or laboratory data. For example, bone mineral density, mechanism and severity of injury, the neurologic condition-related Japanese Orthopedic Association score, the level of fusion, blood loss, and operation duration could not be verified in this database system. Any instances of mortality or perioperative complications after leaving the hospital could not be considered because the database only contains information during hospitalization. In addition, unmeasured confounding factors (e.g., surgeon experience) may have influenced the occurrence of systemic complications and in-hospital deaths. Further research would be needed using database that included more detailed information.

Even with these limitations, we ascertained the novel detailed predictors for surgically treated OVF patients in terms of mortality and perioperative systemic complications with a large national inpatient database.

## Conclusions

We demonstrated the various risk factors related to perioperative systemic complications and in-hospital death in patients who underwent surgery for OVF using a large national inpatient database in Japan. Atrial fibrillation and renal failure as preoperative comorbidities were common factors between perioperative systemic complications and mortality. For patients at particularly high risk of complications, surgical indication should be carefully considered, and the efforts to minimize the perioperative complications are needed.

## Supplementary Information

**Additional file 1.** (XLS 28 kb)

## Data Availability

The datasets used and/or analyzed during the current study are available from the corresponding author on reasonable request.

## Abbreviations

OVF: Osteoporotic vertebral fractures
ADL: Activities of daily living
QOL: Quality of life
DPC: Diagnosis procedure combination
BMI: Body mass index
ICD-10: International classification of diseases, tenth revision
AF: Anterior fusion
PF: Posterior fusion
OR: Odds ratio
CI: Confidence interval
eGFR: Estimated glomerular filtration rate
